# Efficacy Evaluation of Combination Treatment Using Gemcitabine and Radioimmunotherapy with ^90^Y-Labeled Fully Human Anti-CD147 Monoclonal Antibody 059-053 in a BxPC-3 Xenograft Mouse Model of Refractory Pancreatic Cancer

**DOI:** 10.3390/ijms19102979

**Published:** 2018-09-29

**Authors:** Aya Sugyo, Atsushi B. Tsuji, Hitomi Sudo, Mitsuru Koizumi, Yoshinori Ukai, Gene Kurosawa, Yoshikazu Kurosawa, Tsuneo Saga, Tatsuya Higashi

**Affiliations:** 1 Department of Molecular Imaging and Theranostics, National Institute of Radiological Sciences, National Institutes for Quantum and Radiological Science and Technology (QST-NIRS), 4-9-1 Anagawa, Inage-ku, Chiba 263-8555, Japan; sugyo.aya@qst.go.jp (A.S.); sudo.hitomi@qst.go.jp (H.S.); saga@kuhp.kyoto-u.ac.jp (T.S.); higashi.tatsuya@qst.go.jp (T.H.); 2 Department of Nuclear Medicine, Cancer Institute Hospital, 3-8-31 Ariake, Koto-ku, Tokyo 135-8550, Japan; mitsuru@jfcr.or.jp; 3 Research and Development Division, Perseus Proteomics Inc., 4-7-6 Komaba, Meguro-ku, Tokyo 153-0041, Japan; yukai@fujita-hu.ac.jp; 4 Innovation Center for Advanced Medicine, School of Medicine, Fujita Health University, 1-98 Dengakugakubo, Kutsukake-cho, Toyoake, Aichi 470-1192, Japan; gene@fujita-hu.ac.jp (G.K.); kurosawa@fujita-hu.ac.jp (Y.K.); 5 Department of Diagnostic Radiology, Kyoto University Hospital, 54 Shogoinkawahara-cho, Sakyo-ku, Kyoto 606-8507, Japan

**Keywords:** extracellular matrix metalloproteinase inducer, radioimmunotherapy, gemcitabine, combination therapy, pancreatic cancer

## Abstract

The poor prognosis of pancreatic cancer requires the development of more effective therapy. CD147 expresses in pancreatic cancer with high incidence and has a crucial role in invasion and metastasis. We developed a fully human monoclonal antibody (059-053) with high affinity for CD147. Here we evaluated the efficacy of combined treatment using radioimmunotherapy (RIT) with ^90^Y-labeled 059-053 and gemcitabine in a BxPC-3 xenograft mouse model. Expression of CD147 and matrix metalloproteinase-2 (MMP2) in BxPC-3 tumors was evaluated. In vitro and in vivo properties of 059-053 were evaluated using ^111^In-labeled 059-053 and a pancreatic cancer model BxPC-3. Tumor volume and body weight were periodically measured in mice receiving gemcitabine, RIT, and both RIT and gemcitabine (one cycle and two cycles). High expression of CD147 and MMP2 was observed in BxPC-3 tumors and suppressed by 059-053 injection. Radiolabeled 059-053 bound specifically to BxPC-3 cells and accumulated highly in BxPC-3 tumors but low in major organs. Combined treatment using RIT with gemcitabine (one cycle) significantly suppressed tumor growth and prolonged survival with tolerable toxicity. The two-cycle regimen had the highest anti-tumor effect, but was not tolerable. Combined treatment with ^90^Y-labeled 059-053 and gemcitabine is a promising therapeutic option for pancreatic cancer.

## 1. Introduction

Pancreatic cancer is a highly lethal cancer with a 5-year survival rate for all stages of the disease of 8% [1,2]. It is projected to be the second leading cause of cancer death in the USA by 2030 [1,3]. The disease progresses asymptomatically in 80% of patients, and is thus usually detected in an advanced stage with local invasion and/or metastasis, resulting in unresectable cancer. Among the 10–15% of patients who present with resectable disease, 80% experience a relapse [4,5]. Various chemotherapeutic agents are applied for locally advanced and metastatic diseases, but with limited results [2]. Therefore, a new systemic treatment strategy is needed.

CD147 (also known as extracellular matrix metalloproteinase inducer (EMMPRIN) or Basigin) is a 55-kDa transmembrane protein expressed in many types of cancer, including pancreatic cancer [6,7,8]. This protein induces the expression of matrix metalloproteinases (MMPs) and vascular endothelial growth factor [9,10], which are involved in tumor invasion, metastasis, angiogenesis, and proliferation [7,11,12,13]. Therefore, CD147 would be a suitable molecule for targeted therapy against metastatic pancreatic cancer. We developed several fully human monoclonal antibodies against CD147 using a large-scale human antibody library and a screening method that combined living pancreatic cancer cells and organic solvents [14]. Of these antibodies, the antibody 059-053 binds specifically to CD147 with high affinity and induces antibody-dependent cell-mediated cytotoxicity [14,15]. In addition, this antibody, labeled with a positron-emitting radionuclide, Zr-89, accumulates in high levels in CD147-expressing pancreatic cancer xenografts, but in low levels in normal organs and tissues [15].

By substituting Zr-89 with a β-emitting therapeutic radionuclide with the appropriate physical properties, such as Y-90 and Lu-177, the radiolabeled 059-053 can become a promising radioimmunotherapy (RIT) agent for metastatic pancreatic cancer. Pancreatic cancer, however, exhibits resistance to conventional therapy, including radiation [16,17,18], and therefore monotherapy with a radiolabeled antibody is not expected to have sufficient therapeutic effects. This is also suggested by several preclinical studies, including our studies, in which RIT monotherapy with ^90^Y-labeled anti-transferrin receptor antibody in pancreatic cancer mouse models was highly effective in radiosensitive MIAPaCa-2 xenograft tumors, but moderately effective in radioresistant BxPC-3 xenografts [19]. Thus, the development of additional strategies to enhance the therapeutic efficacy of RIT is needed.

Gemcitabine is a standard chemotherapeutic agent widely used as a first-line treatment for patients with advanced pancreatic cancer [2,20,21,22]. Gemcitabine monotherapy and combination therapy with other anticancer drugs are also applied for pancreatic cancer [2,23]. Furthermore, gemcitabine has been shown to act as a radiosensitizer in pancreatic cancer models and patients [20,21,24,25,26,27]. Therefore, combination therapy using RIT with gemcitabine is expected to have a therapeutic effect against pancreatic cancer.

In the present study, we radiolabeled the fully human anti-CD147 monoclonal antibody 059-053 with the γ-emitter In-111 and evaluated the in vitro and in vivo properties using the radioresistant BxPC-3 pancreatic cancer model. We substituted In-111 with the β-emitter Y-90 and evaluated the therapeutic efficacy of ^90^Y-labeled 059-053 alone and in combination with gemcitabine.

## 2. Results

### 2.1. Immunohistochemical Analysis of BxPC-3 Tumors with/without Administration of the Anti-CD147 Antibody 059-053

**Figure 1:**
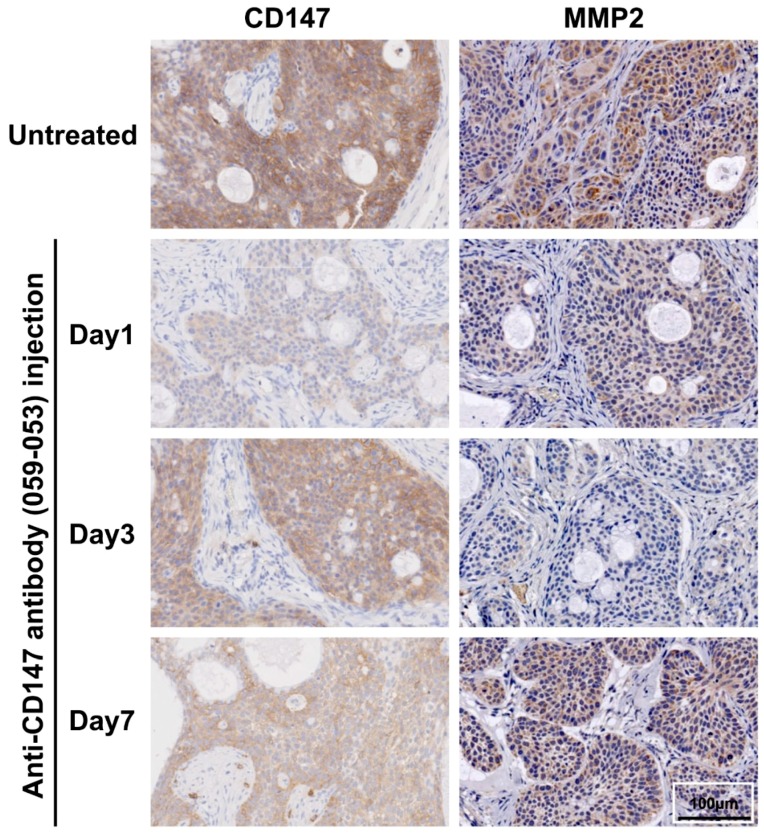
Immunohistochemical staining of CD147 and MMP2 in BxPC-3 xenografted tumors at days 1, 3, and 7 after i.v. injection of intact anti-CD147 antibody 059-053 (25 μg). Sections were immunostained with a goat anti-EMMPRIN/CD147 antibody (diluted 1:500) or an anti-MMP2 polyclonal antibody (diluted 1:200) as the primary antibody.

CD147 and MMP2 were highly expressed in untreated BxPC-3 tumors (Figure 1), whereas MMP9 expression was not detected (data not shown). Injection of the anti-CD147 antibody 059-053 reduced CD147 staining intensity at day 1 and while the intensity increased thereafter, it had not entirely recovered by day 7 (Figure 1). Although MMP2 intensity also decreased at day 1 after 059-053 injection, the weakest intensity was observed at day 3 (Figure 1). The difference in the time at which the weakest staining occurred suggests that the decreased MMP2 expression was induced by the suppression of CD147 expression, which is an inducer of MMP2 [8,28].

### 2.2. Cell Binding and Competitive Inhibition Assays for 059-053

**Figure 2:**
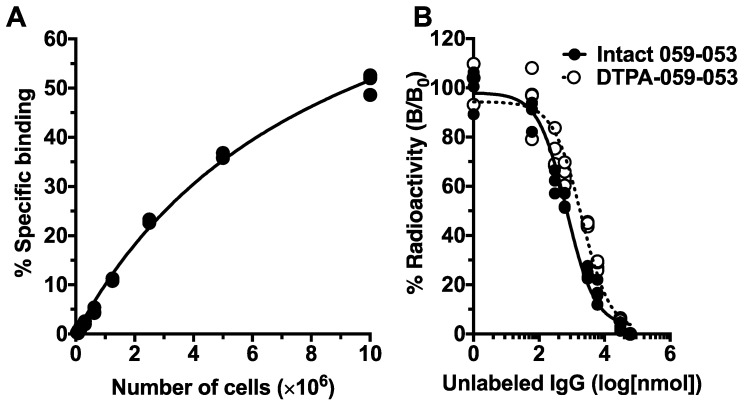
Cell binding and competitive inhibition assays of anti-CD147 antibody 059-053 using BxPC-3 cells. (**A**) Cell binding of ^111^In-labeled 059-053 to BxPC-3 cells. The cell suspensions were incubated with ^111^In-labeled 059-053 on ice for 60 min, and the radioactivity bound to cells after washing was measured. (**B**) Competitive inhibition assay for intact 059-053 (black circles and solid line) and CHX-A″-DTPA-conjugated 059-053 (white circle and dashed line) using BxPC-3 cells. The ^111^In-059-053 was incubated with BxPC-3 cells in the presence of varying concentrations of intact or CHX-A″-DTPA-conjugated 059-053 on ice for 60 min, and the radioactivity bound to the cells was counted after washing.

Cell binding assays showed that ^111^In-labeled 059-053 bound to BxPC-3 cells with a maximum value of 51% at 1 × 10^7^ cells (Figure 2A). On the basis of competitive inhibition assays, the dissociation constants of intact 059-053 and CHX-A″-DTPA-conjugated 059-053 were estimated to be 0.35 and 0.99 nM, respectively (Figure 2B), suggesting that the loss of immunoreactivity by chelator conjugation was limited and acceptable for the following experiments.

### 2.3. Biodistribution of ^111^In-Labeled 059-053 in Mice Bearing BxPC-3 Tumors

Biodistribution experiments of ^111^In-059-053 were conducted in nude mice bearing BxPC-3 subcutaneous tumors from 30 min to day 7 after injection (*n* = 5 each time point) (Table 1). Tumor uptake of ^111^In-059-053 was 1.04 ± 0.16% of the injected dose per gram of tissue (ID/g) and thereafter increased with time until day 4 (16.78 ± 2.61% ID/g), then decreased slightly thereafter (14.98 ± 1.63% ID/g at day 7). Radioactivity in the blood was high, whereas radioactivity in major organs was relatively low (Table 1). Based on the biodistribution data of ^111^In-059-053, the absorbed dose of ^90^Y-labeled 059-053 was estimated. The dose absorbed by BxPC-3 tumors treated with 0.925 MBq, 1.85 MBq, and 3.7 MBq of ^90^Y-059-053 was estimated to be 4.85 Gy, 9.69 Gy, and 19.39 Gy, respectively. Among the major organs, the lungs had the highest absorbed dose (4.35 Gy/MBq; Table 2).

### 2.4. ^90^Y-Labeled 059-053 Treatment in Mice Bearing BxPC-3 Tumors

**Figure 3:**
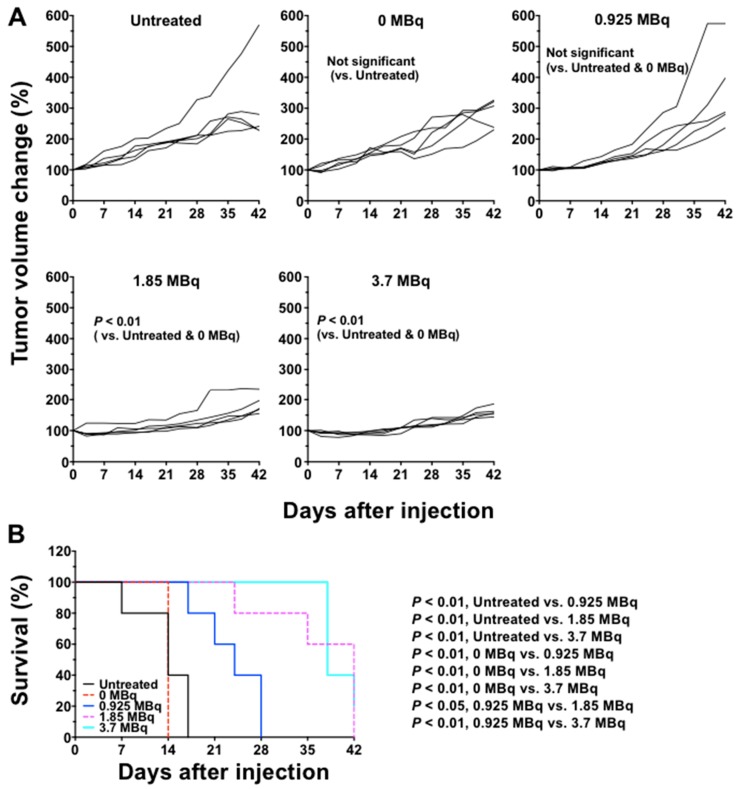
Growth curves of BxPC-3 tumors in mice injected with ^90^Y-hs8001 alone. The injected doses were 0 (antibody only), 0.925, 1.85 and 3.7 MBq of ^90^Y-hs8001. Tumor size was measured at least twice a week. Data are presented as mean ± SD. Individual animal tumor growth curves (**A**) are shown as well as a survival curve (**B**) based on the endpoint of 150% tumor volume.

No significant tumor growth suppression was observed in mice administered unlabeled 059-053 (0 MBq) compared with the untreated group (Figure 3A). Mice treated with 1.85 MBq and 3.7 MBq of ^90^Y-059-053 exhibited significant suppression of tumor growth compared to mice left untreated or treated with 0 MBq (*p* < 0.01; Figure 3A). Tumor growth was not significantly different between mice treated with 0 MBq and 0.925 MBq (Figure 3A). The 1.85-MBq and 3.7-MBq doses suppressed BxPC-3 tumor growth until approximately 2 weeks after the injection, and thereafter the tumor volumes gradually increased (Figure 3A). The Figure 3B shows the survival curves plotted based on an endpoint of 150% of tumor volume. In the untreated, 0-MBq, and 0.925-MBq groups, survival was 0% at day 17, 14, and 28, respectively (Figure 3B). Survival of mice treated with 3.7 MBq of ^90^Y-059-053 was 100% until day 38 and then decreased to 20% at day 42 (end of the observation period), and survival of mice treated with 1.85 MBq was 100% at day 24 and decreased to 0% at day 42 (Figure 3B). Survival was significantly better in mice treated with 1.85-MBq and 3.7-MBq than in mice left untreated, or in those treated with 0 MBq or 0.925 MBq (*p* < 0.01 or *p* < 0.05; Figure 3B). Survival was not significantly different between mice treated with 1.85-MBq and those treated with 3.7-MBq (Figure 3B).

**Figure 4:**
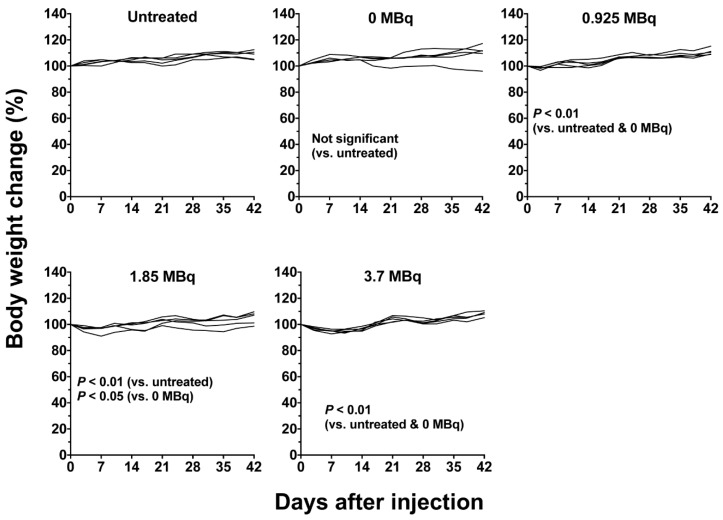
Individual body weight curves of mice bearing BxPC-3 tumors injected with ^90^Y-labeled anti-CD147 antibody 059-053. The injected doses were 0 (intact antibody only), 0.925 MBq, 1.85 MBq and 3.7 MBq of ^90^Y-059-053. Body weight was measured at least twice a week.

Although there was a transient decrease in the body weight of mice treated with ^90^Y-059-053, the body weight recovered (Figure 4). No visible adverse effects, such as diarrhea and dyspnea, were observed at any dose level. Therefore, the dose of 3.7 MBq was used for evaluation of the combined treatments.

### 2.5. Combined Treatment with ^90^Y-Labeled 059-053 and Gemcitabine in Mice Bearing BxPC-3 Tumors

**Figure 5:**
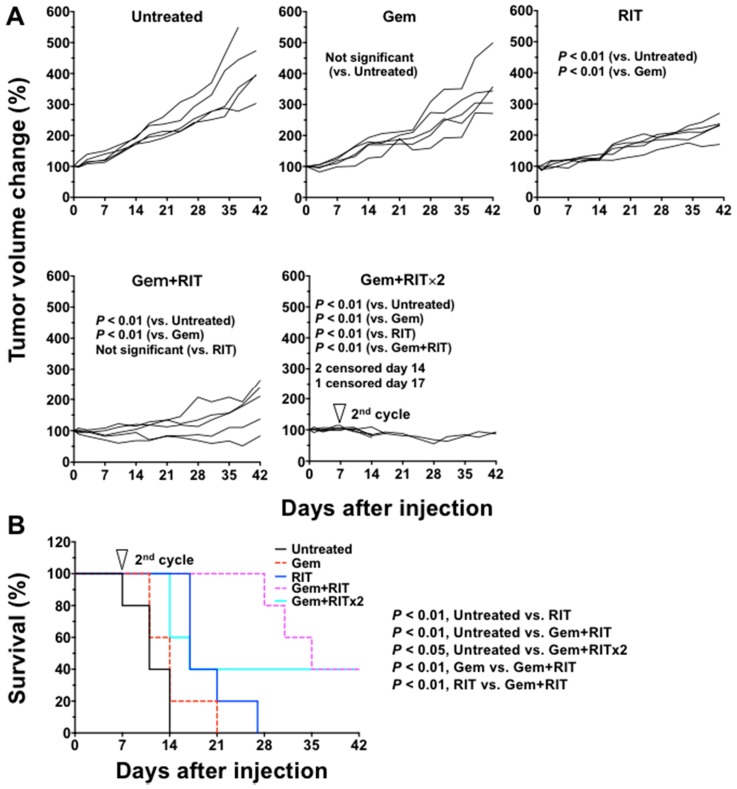
Individual tumor growth curves of BxPC-3 tumors in mice treated with single and combination regimens. The mice were injected with gemcitabine (gem, 240 mg/kg body weight) alone, 3.7 MBq of ^90^Y-labeled anti-CD147 antibody 059-053 (RIT) alone, and the combination of gem plus RIT (one and two cycles) shown in the (**A**). Tumor size was measured at least twice a week. Survival curves were plotted based on the endpoint of 150% tumor volume (**B**).

**Figure 6:**
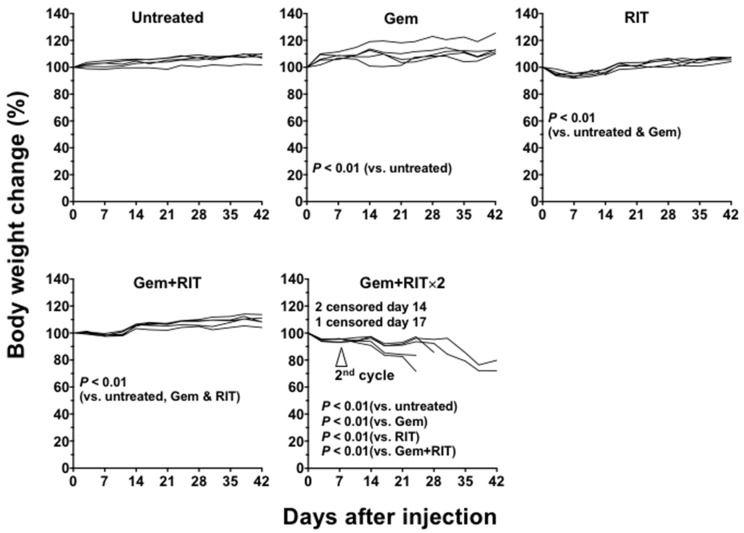
Individual body weight curves of mice bearing BxPC-3 tumors treated with single and combination regimens. The mice were injected with gemcitabine (gem, 240 mg/kg body weight) alone, 3.7 MBq of ^90^Y-labeled anti-CD147 antibody 059-053 (RIT) alone, and the combination of gem plus RIT (one and two cycles). Body weight was measured at least twice a week.

We compared the xenograft tumor size and body weight of mice treated with gemcitabine alone (gem), ^90^Y-059-053 (3.7 MBq) alone (RIT), or combination treatment with gem and RIT in one- and two-cycle dosing regimens (gem + RIT and gem + RIT × 2; Figure 5A). The therapeutic effect did not differ significantly between the gem group and the untreated group. Tumor growth suppression in the RIT, gem + RIT, and gem + RIT × 2 groups was significantly increased compared with that in the untreated and gem groups (*p* < 0.01; Figure 5A). In the gem + RIT group, tumor volume markedly decreased in 2 of 5 mice, but there was no significant difference compared with the RIT group (Figure 5A). In contrast, tumor growth control was significantly better in the gem + RIT × 2 group than in the RIT and gem + RIT groups (Figure 5A). In the gem + RIT × 2 group, however, the body weight in 3 of 5 mice markedly decreased (Figure 6) and severe adverse effects (diarrhea and/or decreased activity) were observed; therefore, these mice were humanely euthanized by an overdose of anesthetic (Figure 5A and Figure 6). Survival curves plotted based on the endpoint of 150% tumor volume are shown in Figure 5B. Survival of the gem + RIT group was 40% at day 42, whereas that of the RIT group was 0% at day 27 (Figure 5B). Survival was significantly different between the gem + RIT and RIT groups (*p* < 0.01; Figure 5B). The gem + RIT × 2 did not prolong the survival compared with RIT groups (Figure 5B).

### 2.6. Histologic Analysis

**Figure 7:**
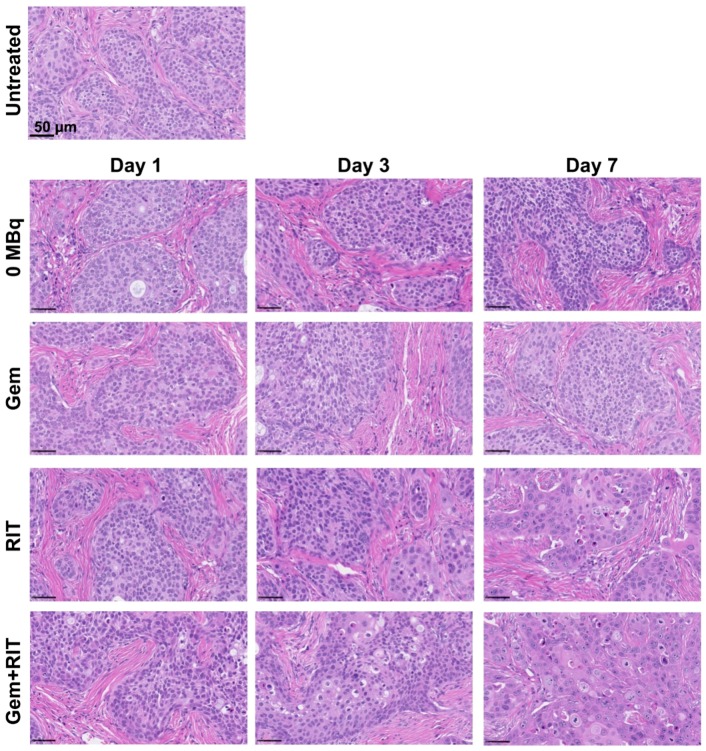
H&E-stained BxPC-3-tumor sections at days 1, 3, and 7 after i.v. injection of intact anti-CD147 antibody 059-053 (0 MBq), gemcitabine (gem, 240 mg/kg body weight) alone, 3.7 MBq of ^90^Y-labeled 059-053 (RIT) alone, and the combination of gem plus RIT (one-cycle regimen).

**Figure 8:**
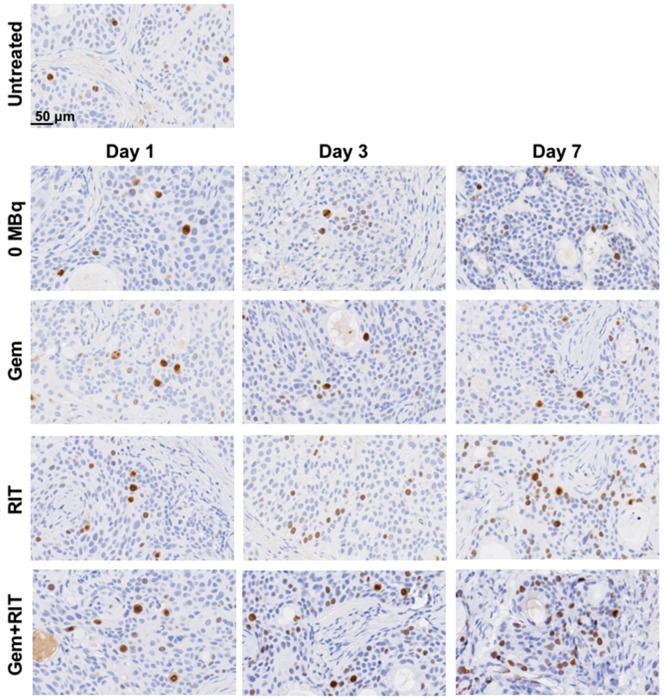
Ki-67-stained BxPC-3-tumor sections at days 1, 3, and 7 after i.v. injection of intact anti-CD147 antibody 059-053 (0 MBq), gemcitabine (gem, 240 mg/kg body weight) alone, 3.7 MBq of ^90^Y-labeled 059-053 (RIT) alone, and the combination of gem plus RIT (one-cycle regimen). The Ki-67 antigen was detected using an anti-human Ki-67 polyclonal antibody as the primary antibody (diluted 1:100).

Hematoxylin and eosin-stained sections of BxPC-3 tumors treated with intact anti-CD147 antibody 059-053 (0 MBq) and gemcitabine exhibited no significant differences compared with those of untreated tumors for up to 7 days after injection (Figure 7). In contrast, sections of tumors treated with 3.7 MBq of ^90^Y-labeled 059-053 (RIT) alone or a combination of RIT with gemcitabine (one-cycle regimen) exhibited cellular hypertrophy at day 7 after the injection (Figure 7). Ki-67-stained sections of BxPC-3 tumors showed no marked difference of cell proliferation by treatments (Figure 8).

### 2.7. Tumor Growth Curve Following External Beam Radiation with X-rays with or without Gemcitabine

**Figure 9:**
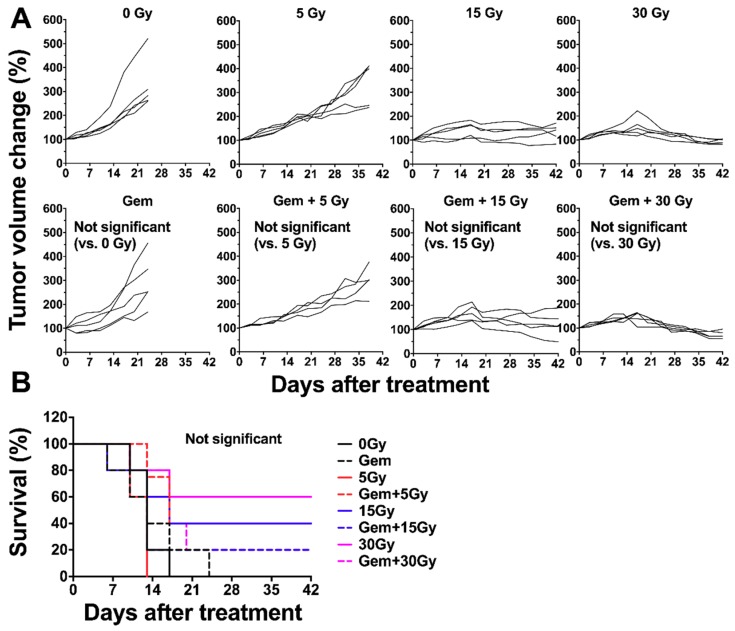
Individual tumor growth curves of BxPC-3 tumors treated with X-ray radiation with or without gemcitabine (gem). 0 Gy, gem (240 mg/kg body weight) alone, 5 Gy, gem plus 5 Gy, 15 Gy, gem plus 15 Gy, 30 Gy, and gem plus 30 Gy shown in the (**A**). Tumor size was measured at least twice a week. Survival curves were plotted based on the endpoint of 150% tumor volume (**B**).

**Figure 10:**
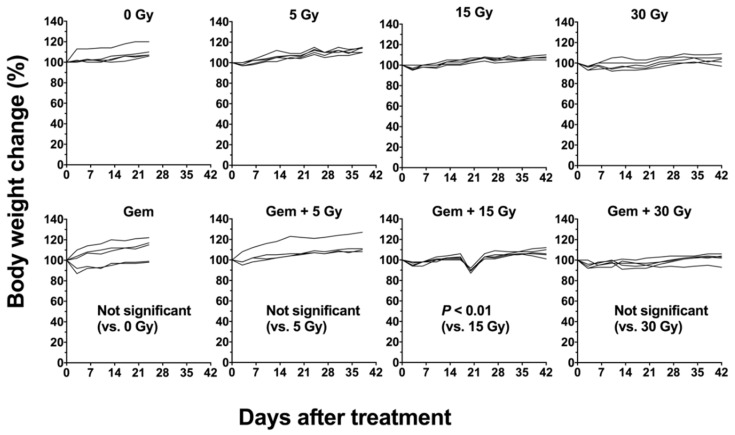
Individual body weight curves of mice bearing BxPC-3 tumors treated with X-ray radiation with or without gemcitabine (gem). 0 Gy, gem (240 mg/kg body weight) alone, 5 Gy, gem plus 5 Gy, 15 Gy, gem plus 15 Gy, 30 Gy, and gem plus 30 Gy. Body weight was measured at least twice a week.

Effects of combination treatment with gemcitabine and external beam radiation (instead of ^90^Y-labeled antibody) were examined in BxPC-3 tumor-bearing mice. Although there is no statistically significant difference, the combination of X-ray radiation with gemcitabine tended to show significantly stronger tumor suppressing effects than radiation alone at the corresponding dose (Figure 9A). No significant prolonged survivals by adding gemcitabine were also observed (Figure 9B). There is no significant difference in body weight between radiation alone and the combination at the corresponding dose, except for the 15-Gy-treatment groups (Figure 10). The mice treated with the combination of 15-Gy radiation with gemcitabine showed a transient body weight loss around day 21 because of a drinking-water accident.

## 3. Discussion

Most patients with pancreatic cancer have an inoperable disease with invasion and/or metastasis, and current systemic therapies are not sufficiently effective for these patients [2,29]. The development of an effective systemic therapy to improve the prognosis of pancreatic cancer remains a challenge. RIT is a candidate therapeutic option, and several studies including ours have reported significant anti-tumor effects of RIT in mouse models of pancreatic cancer [19,30]. RIT exhibits significant effects in pancreatic cancer xenografts that are sensitive to therapy, including radiation, but the effects are limited in xenografts that are resistant to radiation [19,30], suggesting the need to enhance the efficacy of RIT for refractory cancer. In the present study, we evaluated the efficacy of combined treatment using RIT and gemcitabine, which is the standard drug used to treat pancreatic cancer as well as a radiosensitizer, in nude mice bearing BxPC-3 tumors that are resistant to radiation [19].

The present study targeted CD147 because of the reported high incidence (87%) of CD147 expression in pancreatic cancer [6], and its crucial role in invasion and metastasis [7]. We developed a fully human anti-CD147 monoclonal antibody, 059-053, that exhibits high affinity and antibody-dependent cell-mediated cytotoxicity [14]. Our previous study showed that radiolabeled 059-053 accumulates in high levels in MIAPaCa-2 xenograft pancreatic cancer tumors with high CD147 expression, and BxPC-3 cells have low CD147 protein expression in vitro [15], although we observed discrepancies between in vitro and in vivo expression of another antigen in our previous study [31]. In the present study, in vivo CD147 expression analysis revealed high expression in BxPC-3 tumors, suggesting the possibility of high tumor uptake of the antibody 059-053 in BxPC-3 tumors.

In our previous study, the antibody 059-053 was radiolabeled with Zr-89 for positron emission tomography [15]. To label an antibody with a therapeutic radionuclide Y-90 and its surrogate radionuclide In-111 requires a DTPA or DOTA analog-conjugated antibody, and the deferoxamine analog used for Zr-89 labeling is not suitable. In the present study, the DTPA analog CHX-A″-DTPA was used for labeling In-111 and Y-90. An in vitro binding study of ^111^In-labeled antibody 059-053 indicated that the affinity after CHX-A″-DTPA conjugation was acceptable for further application in in vivo experiments.

The biodistribution of ^111^In-labeled antibody 059-053 in mice bearing BxPC-3 tumors showed high tumor uptake (peak value, 16.78 ± 2.61% ID/g), comparable to the tumor uptake of ^89^Zr-labeled 059-053 in MIAPaCa-2 tumors with high CD147 expression [15]. As mentioned above, the CD147 expression was markedly lower in cultured BxPC-3 cells than in MIAPaCa-2 cultured cells [15], whereas expression in BxPC-3 tumors was similar to that in MIAPaCa-2 tumors. The expectation of high tumor uptake of the radiolabeled 059-053 in BxPC-3 tumors was confirmed by the present biodistribution study.

The absorbed dose of ^90^Y-labeled 059-053 was estimated based on the biodistribution data of ^111^In-labeled 059-053. The absorbed dose to BxPC-3 tumors was 5.24 Gy/MBq and 19.38 Gy when injected with 3.7 MBq of ^90^Y-059-053, which was the highest amount of radioactivity administered in the present study. The absorbed doses to the major organs were also estimated and the highest was found in the lung (4.35 Gy/MBq) where the absorbed dose after injection of 3.7 MBq of ^90^Y-labeled 059-053 was calculated to be 16.10 Gy. This value is considered to be tolerable in mice based on a lung tolerance dose of 17.5 Gy of external beam radiation [32] and the tolerability observed in several preclinical studies of RIT, including our previous studies [15,30,33]. The absorbed doses to the other organs were also tolerable for single dosing. In combined treatment with gemcitabine, however, especially in the two-cycle regimen described below, the tolerability should be evaluated in addition to the anti-tumor effects observed in the present study.

For evaluation of the treatment effects, we first evaluated the efficacy of ^90^Y-labeled 059-053 alone. Significant tumor suppression and prolonged survival were observed after administering 1.85 MBq and 3.7 MBq of ^90^Y-059-053, and these doses were tolerable in mice with only transient weight loss. Next, using 3.7 MBq as the dose for RIT, we conducted combined treatment studies with gemcitabine. The one-cycle regimen of combined treatment significantly reduced tumor size, although the anti-tumor effects were varied and no significant difference was detected compared with RIT alone. The survival analysis, however, demonstrated significantly prolonged survival in mice treated with the one-cycle regimen compared to mice treated with RIT alone. Considering that a single treatment of gemcitabine did not induce a significant tumor growth delay and survival benefit, the augmented growth delay and better survival observed with combined treatment seems to be induced by the radiation-sensitizing effect of gemcitabine, and not by its cytotoxic effect. The results were similar for the combined treatments with external beam radiation and gemcitabine. Although the two-cycle regimen of RIT with gemcitabine showed a greater anti-tumor effect than the other treatments, the regimen was not tolerable, indicating the need to optimize the dose and interval.

As mentioned above, the present study demonstrated that combined treatment using ^90^Y-labeled 059-053 RIT with gemcitabine is a potentially new therapeutic option, but its efficacy toward a cure for refractory pancreatic cancer requires improvement. There are several potential strategies for improvement, as follows. First, as mentioned above, further studies are required to optimize dosing regimens such as the gemcitabine dose and administration interval. The dose of gemcitabine used in the present study had a radiation-sensitizing effect but a negligible cytotoxic effect. The toxicity of a high dose of gemcitabine is severe, and dose escalation would be limited. Recently, several gemcitabine-loaded nanoparticles were developed that exhibit greater efficacy and lower toxicity than standard gemcitabine [33]. Using these drug delivery systems for ^90^Y-labeled 059-053 in combination with RIT may be more effective. Alternatively, longer inter-dose intervals in a multi-cycle regimen could reduce radiation toxicity. In fact, the intervals of most radionuclide therapies, such as ^131^I-MIBG and ^177^Lu-DOTATATE, are approximately 8 weeks in most clinical settings with a reasonable tradeoff between toxicity and therapeutic response, although widely different intervals have been used [34,35]. Second, replacing the β-emitter Y-90 with a more cytotoxic radionuclide such as an α-emitter may be more effective. Recently, a prostate-specific membrane antigen-targeted agent, PSMA-617, labeled with the α-emitter Ac-225 showed a complete response in patients with metastatic prostate cancer [36]. The half-life of Ac-225 is 10 days and suitable for RIT; in fact, clinical trials with ^225^Ac-labeled antibody have already been conducted [37]. Third, repeated administration of the unlabeled anti-CD147 antibody 059-053 following combination therapy with ^90^Y-labeled 059-053 and gemcitabine has the potential to be more effective because the antibody exhibits antibody-dependent cell-mediated cytotoxicity [14] and 059-053 administration decreases CD147 and MMP2 expression, as shown in the present study. The 059-053 antibody is a fully human monoclonal antibody and would be suitable for repeated administration with low immunogenicity unlike antibodies containing murine proteins [38].

In summary, combined treatment using a ^90^Y-labeled fully human anti-CD147 antibody 059-053 with gemcitabine significantly suppressed tumor growth and prolonged survival in a BxPC-3 xenograft mouse model of refractory pancreatic cancer. Further efforts should be made to enhance the anti-tumor effects with tolerable toxicity. The combination of ^90^Y-labeled 059-053 and gemcitabine is a promising therapeutic option and our findings warrant further studies to optimize dosing regimens to improve the efficacy with acceptable toxicity.

## 4. Materials and Methods

### 4.1. Cells

The human pancreatic cancer cell line BxPC-3 was obtained from ATCC (Manassas, VA, USA). The cells were maintained in RPMI1640 medium (Wako Pure Chemical Industries, Osaka, Japan) supplemented with 10% fetal bovine serum (Sigma, St. Louis, MO, USA) in a humidified incubator maintained at 37 °C with 5% CO_2_.

### 4.2. Subcutaneous Tumor Mouse Model

The Animal Care and Use Committee of the National Institute of Radiological Sciences approved the protocol for the animal experiments (13-1023, 27 March 2014), and all animal experiments were conducted by following the institutional guidelines regarding animal care and handling. Male BALB/c-nu/nu mice (5 weeks old, CLEA Japan, Tokyo, Japan) were maintained under specific pathogen-free conditions. Mice were inoculated subcutaneously with BxPC-3 cells (4 × 10^6^) in the left thigh under isoflurane anesthesia.

### 4.3. Immunohistochemistry of CD147, MMP2, and MMP9 in BxPC-3 Tumors

Tumors (*n* = 2/time-point) were sampled at days 1, 3, and 7 after intravenous injection of intact 059-053 (25 µg) into tumor-bearing mice, fixed in 10% (*v*/*v*) neutral buffered formalin, and embedded in paraffin for sectioning. Untreated tumors were used as a control. Sections (1-µm thick) were immunostained with a goat anti-EMMPRIN/CD147 antibody (AF972, R&D Systems, Minneapolis, MN, USA, diluted 1:500) as described previously [15]. MMP2 and MMP9 were detected using anti-MMP2 and anti-MMP9 polyclonal antibodies, respectively, as the primary antibody (Sigma-Aldrich Japan, Tokyo, Japan; diluted 1:200 and 1:250, respectively). Nonspecific binding was blocked by Protein Block Serum-Free reagent (Agilent Technologies, Santa Clara, CA, USA). The specimens were incubated overnight at 4 °C with the primary antibody. The secondary antibody (Dako EnVision+ system, horseradish peroxidase-labeled polymer for anti-rabbit, Agilent Technologies (Santa Clara, CA, USA) was applied for 30 min at room temperature and then visualized with a diaminobenzidine staining reagent (Dako Liquid DAB+ Substrate Chromogen system, Agilent Technologies).

### 4.4. Radiolabeling of Antibody

The antibody 059-053 was conjugated with *p*-SCN-Bn-CHX-A″-DTPA (DTPA; Macrocyclics, Dallas, TX, USA) as previously described [19], and the DTPA-conjugated antibody was purified using a Sephadex G-50 (GE Healthcare, Little Chalfont, UK) column (700× *g* for 2 min). The conjugation ratio of DTPA to antibody was estimated to be approximately 1.5 based on cellulose acetate electrophoresis. For In-111 labeling (three experiments), 50 μg DTPA-conjugated antibody was mixed with 0.74 MBq ^111^InCl_3_ in 0.5 M acetate buffer (pH 6.0) and the mixture was incubated for 30 min at room temperature. For Y-90 labeling (two experiments), 100 or 222 μg DTPA-conjugated 059-053 was mixed with 88.8 or 133.2 MBq ^90^YCl_3_ in 0.5 M acetate buffer (pH 6.0), respectively, and the mixture was incubated for 30 min at room temperature. The radiolabeled antibodies were separated from free radionuclides on a Sephadex G-50 column (700× *g* for 2 min). The labeling yields of ^111^In-labeled 059-053 were 77% to 79%, and those of ^90^Y-labeled 059-053 were 82% and 76%. The radiochemical purities of all the labeled antibodies exceeded 97%. The specific activities of ^111^In-059-053 ranged between 11 and 12 kBq/μg, and those of ^90^Y-059-053 were 728 and 456 kBq/μg.

### 4.5. *In Vitro* Assays for Antibody 059-053

Cell binding and competitive inhibition assays were conducted as previously described [39]. Briefly, in cell binding assays, 3 to 4 days after seeding, we detached BxPC-3 cells and prepared various concentrations of cell suspensions (1.0 × 10^7^, 5.0 × 10^6^, 2.6 × 10^6^, 1.3 × 10^6^, 6.3 × 10^5^, 3.1 × 10^5^, 1.6 × 10^5^, 7.8 × 10^4^, and 3.9 × 10^4^ in 1 mL phosphate-buffered saline (PBS) with 1% bovine serum albumin). The suspensions were incubated with ^111^In-labeled 059-053 on ice for 60 min, and the radioactivity bound to cells after washing was measured using a γ counter (Aloka, Tokyo, Japan). In competitive inhibition assays, ^111^In-059-053 was incubated with BxPC-3 cells in the presence of varying concentrations of intact or DTPA-conjugated 059-053 (0, 0.5, 1, 5, 10, 50, and 100 ng/μL) on ice for 60 min, and the radioactivity bound to the cells was counted after washing. The dissociation constant was estimated using GraphPad Prism 6 software (GraphPad Software, La Jolla, CA, USA).

### 4.6. Biodistribution of ^111^In-Labeled 059-053 and Absorbed Dose Estimation for ^90^Y-Labeled 059-053

When subcutaneous tumors reached a diameter of approximately 8 mm (approximately 5 weeks after inoculation), mice (*n* = 5/time-point) were intravenously injected with 37 kBq of ^111^In-059-053. The total injected protein dose was adjusted to 25 µg per mouse by adding intact 059-053. At 30 min, and 1, 2, 4, and 7 days after injection of ^111^In-059-053, the mice were euthanized by isoflurane inhalation, and blood was obtained from the heart. The tumors and major organs were removed and weighed, and the radioactivity was measured using a γ counter (Aloka). The data were expressed as the percentage of injected dose per gram of tissue (% ID/g) normalized to a mouse with a body weight of 20 g. The absorbed dose for ^90^Y-059-053 was estimated using the area under the curve of each organ based on the biodistribution data of ^111^In-059-053 and the mean energy emitted per transition of Y-90, 1.495 × 10^−13^ Gy kg (Bq·s)^−1^ [40] as described previously [32].

### 4.7. Therapeutic Experiments of ^90^Y-Labeled 059-053 with or without Gemcitabine

When subcutaneous tumors reached a diameter of approximately 8 mm (approximately 5 weeks after inoculation), therapeutic experiments were conducted. For ^90^Y-059-053 alone, mice (*n* = 5/dose) bearing subcutaneous BxPC-3 tumors were injected with 0.925 MBq, 1.85 MBq, or 3.7 MBq of ^90^Y-059-053 into a tail vein. The protein dose was adjusted to 25 µg for each preparation by adding intact 059-053. As a control, mice were intravenously injected with the intact antibody 059-053 (25 µg protein/mouse) or PBS (defined as untreated). For the combination treatment experiment, mice (*n* = 5/group) bearing subcutaneous BxPC-3 tumors were injected with PBS, gemcitabine (240 mg/kg body weight) alone, 3.7 MBq of ^90^Y-059-053 alone, or a combination of ^90^Y-059-053 and gemcitabine (one cycle or two cycles once a week) into a tail vein. Gemcitabine (S1149, Selleck, Houston, TX, USA) solubilized in sterile saline was intravenously administered 24 h before the ^90^Y-059-053 injection. In the two-cycle regimen, the mice were administered again at 7 days after 1st injection. Body weight and tumor size were measured at least twice a week for 42 days. When the tumor reached 15 mm in diameter, the mouse was euthanized humanely by isoflurane inhalation. Tumor volume (mm^3^) was calculated as (length × width^2^)/2. The tumor volume data were normalized by the volume at day 0 and analyzed by two-way repeated-measures analysis of variance. Survival was plotted based on the time to progression of the tumor to 150% of the baseline size and analyzed by the log-rank test.

### 4.8. External Beam Radiation with X-rays with or without Gemcitabine

BxPC-3 tumors (*n* = 5/dose) were irradiated with 0 Gy, 5 Gy, 15 Gy, or 30 Gy of X-rays at a rate of 4.5 Gy/min with a TITAN-320 X-ray generator (Shimadzu, Kyoto, Japan). In the combination treatment with X-ray radiation and gemcitabine, gemcitabine (240 mg/kg body weight) was intravenously administered 24 h before radiation. Body weight and tumor size were measured at least twice a week for 42 days. When the tumor reached 15 mm in diameter, the mouse was euthanized humanely by isoflurane inhalation. Tumor volume (mm^3^) was calculated as (length × width^2^)/2. The tumor volume data were normalized by the volume at day 0 and analyzed by two-way repeated-measures analysis of variance.

### 4.9. Histologic Analysis of Tumors

As a separate experiment, tumor samples (*n* = 3 /time-point) were extirpated at days 1, 3, and 7 after intravenous injection of intact anti-CD147 antibody 059-053 (0 MBq), gemcitabine alone, 3.7 MBq of ^90^Y-labeled 059-053 (RIT) alone, or the combination of RIT with gemcitabine. Untreated tumors (*n* = 3) were used as a control. The tumors were fixed in 10% (*v/v*) neutral buffered formalin and embedded in paraffin for sectioning. Sections (5-µm thick) were stained with hematoxylin and eosin. The Ki-67 antigen was detected using an anti-human Ki-67 polyclonal antibody (Dako Denmark, Glostrup, Denmark, diluted 1:100) as described previously [41].

## Figures and Tables

**Table 1:** Biodistribution of ^111^In-labeled anti-CD147 antibody 059-053 in mice bearing BxPC-3 tumors.

	30 min	Day 1	Day 2	Day 4	Day 7
Blood	53.98 ± 2.85	27.97 ± 2.85	25.62 ± 1.78	20.39 ± 3.11	13.10 ± 2.97
Lung	17.12 ± 2.77	10.44 ± 1.58	9.51 ± 0.98	7.96 ± 2.28	6.18 ± 0.44
Liver	8.96 ± 0.85	5.89 ± 0.49	6.22 ± 0.60	6.56 ± 1.59	5.64 ± 1.40
Spleen	6.67 ± 1.57	4.59 ± 0.57	4.56 ± 0.48	4.05 ± 0.88	3.68 ± 0.21
Pancreas	2.67 ± 0.82	2.05 ± 0.22	1.97 ± 0.20	1.58 ± 0.32	1.15 ± 0.22
Intestine	2.29 ± 0.29	2.20 ± 0.32	2.18 ± 0.23	1.82 ± 0.37	1.34 ± 0.32
Kidney	10.51 ± 0.52	5.97 ± 0.64	6.03 ± 0.53	4.90 ± 0.77	3.66 ± 0.50
Muscle	0.71 ± 0.17	1.35 ± 0.21	1.40 ± 0.21	1.24 ± 0.25	0.97 ± 0.07
Bone	2.75 ± 0.29	2.09 ± 0.29	2.34 ± 0.44	1.98 ± 0.45	1.73 ± 0.10
BxPC-3	1.04 ± 0.16	9.23 ± 0.67	16.13 ± 0.92	16.78 ± 2.61	14.98 ± 1.63

Data are expressed as % ID/g ± SD.

**Table 2:** Absorbed dose of ^90^Y-labeled anti-CD147 antibody 059-053 to major organs and BxPC-3 tumor.

Organ	Gy/MBq
Lung	4.35
Liver	2.81
Spleen	1.99
Pancreas	0.82
Intestine	0.87
Kidney	2.65
Muscle	0.51
Bone	0.94
BxPC-3	5.24
